# The insufficiency of the Malaysian contact tracing app from the perspective of Chinese tourists: preparing for international tourism in the post-COVID-19 world

**DOI:** 10.1016/j.heliyon.2022.e12154

**Published:** 2022-12-09

**Authors:** Xiaocong Jiang, Ahmad Edwin Mohamed

**Affiliations:** a School of Tourism, Hospitality and Event Management, Universiti Utara Malaysia, Sintok, Malaysia; b School of Business, Institute of Vocational Technology, SIP, Suzhou, China

**Keywords:** Mysejahtera, Contact-tracing app, International tourism, COVID-19, Resumption

## Abstract

Understanding tourists' feedback on using Mysejahtera is critical for tourism recovery in these destinations, and even more so for countries like Malaysia and China, where national Contact Tracing Applications (CTA) are mandatory. However, Previous surveys on CTA use have mainly focused on voluntary CTA users, using qualitative research methods. In this research, Chinese overseas students in Malaysia are included as the reference group, and Chinese tourists with experiences traveling overseas are put into the experimental group. A total of 890 questionnaires were collected and taken as the original data to carry out the Chi-squared and Mann-Whitney U tests. Meanwhile, the experiment implemented a multiple linear regression mechanism to explore the variables that may improve the app Mysejahtera, with further analysis being conducted. According to the results, language issues are the most significant barrier to Chinese visitors using MySejahtera; the inability to register with a Chinese mobile phone number and the need to register a permanent address in Malaysia have a significant negative impact on the use of MySejahtera; and visitors' trust in science positively related to MySejahtera use.

## 1 Introduction

COVID-19 has become a worldwide pandemic since its first outbreak in China. It has been almost two years since the World Health Organization declared a pandemic on 11 March 2020 (UNWHO, 2020b). Although vaccination is imposed on most populations globally (Salathé et al., 2020), controlling and preventing COVID-19 remains challenging for many countries. Screening, limiting the population flow, and tracing contacts are considered the basic regulating approaches in COIVD-19 prevention (UNWHO, 2020a). Limited screening resources and a lack of experts have resulted in many countries' inability to full-city screening (CGTN, 2021a; Esbin et al., 2020), a measure that has been operated successfully in China. Many governments turned to traffic flow restrictions or city blocking to control Covid-19 (Chiu et al., 2020; Rocklöv et al., 2020). Finding a powerful screening system to acquire necessary scientific data to tackle COVID-19 remains vital since many countries have loosened restrictions (Abd-Alrazaq et al., 2020). Meanwhile, the public is also eager to have a plausible method to know Covid contacts’ information and travel history (Abd-Alrazaq et al., 2020). Given the situation, the Malaysian government implemented the contact tracing application (CTA) named Mysejahtera to halt the increasing Covid numbers. Mysejahtera featured essential functions, including tracing Covid contacts, delivering COVID-19 prevention information, and Vaccination updates. The vaccinees can also show the vaccination history through the user interface. Installing the CTA depends merely on people's willingness in many countries, but Malaysia is among the few countries that made the apps mandatory for its citizens (Mozur et al., 2020). According to the research by Chan et al. (2021), 79.1% users in Malaysia have installed the application. Mysejahtera meets more than 60% of the requirements, which Hinch et al. (2020) claimed could effectively prevent COVID. Since August 2020, the Malaysian government has required everyone to scan codes using Mysejahtera while entering public spaces such as public transportation, malls, tourism spots, and hotels (CodeBlue, 2020). While foreign tourists entering the Malaysian border also need to use Mysejahtera to abide by the COVID-19 prevention terms. The Malaysian international tourism industry has suffered significant losses due to the pandemic. Since 2020, total arrival has decreased 83.4%, equivalent to 4.3 million, and tourism receipts fell to 13.7 billion in 2021 from 92.6 billion Malaysian ringgit. Prior to the COIVD pandemic, Chinese tourists played an essential role in Malaysia's international tourism industry. The current Mysejahtera is incompatible with China's health code, so it cannot assure more efficient contact tracing (Altmann et al., 2020). Instead, the Malaysian government has regulated that tourists can only use Mysejahtera to apply health codes and scan before entering the border. In other words, ensuring Chinese tourists apply Mysejahtera strongly impacts reviving Malaysia's international tourism industry.

Most researchers pointed out that visitors should install CTA voluntarily (BASU, 2020), a significant issue that recent studies focus on. Studies on mandatory usage mainly discuss residents' problems using this application (Akinbi et al., 2021). Currently, no studies have been carried out on improving the CTA from the perspective of international tourists. For studies focusing on MySejahtera specifically, most analyze the issue from the residents' perspective. As for the application itself, the most frequently studied issues include software safety, its ease of use, application scenarios, and other possible technological improvements. All these studies ignored problems that may face international tourists, such as language barriers, the inability to register the software, and time it takes to review vaccination certificates.

This work fills the gap mentioned above by taking the Chinese tourists as the objects. Whether there is room for improvement in registering and using the app is explored. In addition, this work investigated to what extent the tourists trust the local government and science and whether CTA helps control the pandemic. In terms of the study method, previous research on CTA is primarily qualitative studies, and there are insufficient quantitative ones. This research appointed the Chinese overseas students as the reference group and the tourists with overseas traveling experience as an experimental group to proceed with online research. It used statistics to analyze those two groups' differences and explored the experimental groups' complaints regarding deficiencies in the app Mysejahtera. The study also overviewed the Chinese tourists' sentiments over the Malaysian health code application. Furthermore, it helped the authority or regions that impose mandatory health code application improve their related technology and methods to prepare for the new global tourists boom to enter their country.

To achieve the purposess of the present study the following questions were raised:

1 What is the experience of using mandatory CTAs like from a Chinese tourist's perspective?
2 Can compulsory CTAs be studied through a quantitative empirical research approach?

## 2 Literature review

### 2.1 Contact tracing apps (CTA)

CTA enables the country that uses it to recover from the pandemic faster (Bibbiani, 2020), and therefore many countries utilized different technological approaches to design CTA that adapts to their own nations' conditions. However, research on these applications mostly takes residents as objects (Howell and Potgieter, 2021). Among the current articles about CTAs, Urbaczewski and Lee (2020) researched multiple countries' CTAs and articulated those apps' technical features and adoption rates. Altmann et al. (2020) examined the adoption rates of the local CTA regarding the locals. Yasaka et al. (2020) have conducted research analyzing the CTAs validity. The issue of privacy protection in using CTA is also the focus of current research (BASU, 2020).

Konidaris et al. (2021) pointed out that CTA plays a positive role in visitors' health, and relevant studies have been carried out in countries where CTA is used voluntarily. Most existing research on such issues focuses on privacy and security in use, visitors' willingness to use, and public value in use (Fox et al., 2021; Ukpabi et al., 2020; Gerli et al., 2021). Park et al. (2021) analyzed how CTA can be used to screen visitors for high-risk situations when entering confined indoor spaces, such as cafes and restaurants while protecting their privacy.

### 2.2 Mandatory CTA

Although Osmanlliu et al. (2021) argue that voluntary or mandatory is not the primary determinant of the CTAs effectiveness, and the ability to use them correctly and efficiently is fundamental, most countries still choose to use voluntary CTAs, believing that such APPs should be installed voluntarily by the user (BASU, 2020). Only countries such as China, Malaysia, South Korea, and India have used Mandatory CTA for pandemic control (Howell and Potgieter, 2021; Urbaczewski and Lee, 2020). Most existing literature on such issues has been conducted through qualitative research methods, focusing on local population and precautions (Akinbi et al., 2021; HKSAR, n.d.). Other researchers have focused more on privacy protections, with most concluding through qualitative methods that mandatory CTAs violate users’ privacy rights (Shahroz et al., 2021; Wiertz et al., 2020; Rowe, 2020); while Lucivero et al. (2020) on the other hand, argue that mandatory CTAs are more ethical. Most existing studies on mandatory CTA have been conducted on residents or national travelers, and there are no studies involving international visitors.

### 2.3 CTA of Malaysia: Mysejahtera

Previous articles have mainly studied Mysejahtera from the residents' perspective using a qualitative research method. Chan et al. (2021) analyzed the intended use of the Mysejahtera, and Amin et al. (2020) studied the safety and operability of the Mysejahtera from a technological background. Abdali et al. (2021) brought up optimization proposals on Mysejahtera regarding its technology. The current articles have analyzed Mysejahtera's privacy and ethical implications behind the scenes and articulated its propelling impact on Malaysia's domestic tourism industry (Aji et al., 2021; Abeler et al., 2020; Vokinger et al., 2020). Some academics claim that the vaccination status on Mysejahtera can supplant the role of the vaccine passport, which could help revive Malaysia's international tourism industry (Pavli and Maltezou, 2021; Sharun et al., 2021; Sotis et al., 2021). There is no research about visitors using the host countries' health code services in the current literature inventory. The formerly conducted study also neglected to discuss the discrepancy between the application scenarios of Mysejahtera and the users' cognitive ability and resources. Moreover, there have not been articles from tourists' perspectives that discuss the hidden issues that might occur and affect the tourists' daily activities, especially when they cannot apply the imposed applications.

### 2.4 Chinese tourists

Before the outbreak of the pandemic, Chinese tourists once reached 3.11 million, accounting for up to 11.9% of overall foreign tourists in 2019, and tourist expenditure mounted to 86.1 billion ringgit, which made up for 17.8% of the overall foreign tourist expenditure (Department of Statistics, Malaysia, 2020). However, due to political or cultural reasons, Chinese tourists have unique aesthetic profiles and perspectives, and their market characteristics and preferences are distinct from those of other international tourists (Ateljevic and Doorne, 2000; Liu et al., 2019). Firstly, the emphasis on collectivism is a distinctive feature of Chinese culture (Fan, 2000), primarily guided by governmental norms and obligations regarding behavior (Ravlin et al., 2012). Western visitors are less subject to the constraints associated with their governments and are also more individualistic (Dai et al., 2019). Second, unlike Western societies, Chinese residents are subject to more governmental coercion (Liu and Graham, 2021). Since the beginning of the COVID-19 pandemic, the Chinese government has mandated the use of CTAs by residents (Howell and Potgieter, 2021), with robust laws and regulations governing the mandatory use (Liu and Graham, 2021). Although the epidemic has improved worldwide since 2021, the Chinese government still subjectively defines all countries/regions outside China, except Macau, as high-risk areas. The only outbound destination for Chinese residents was Macau, which indirectly contributed to a surge in tourism to the region during the pandemic (Macau Government Tourism Office, 2021), while the number of people traveling to other destinations fell sharply. Although Chinese residents have embraced mandatory CTAs in their own country (Lyu and Carroll, 2022), no studies have been conducted to examine the experiences of Chinese residents using mandatory CTAs in other countries from a visitor perspective.

### 2.5 Research overview

Firstly, most of the existing studies discuss the potential privacy issues, technical issues, and acceptability of CTAs from the perspective of the country's residents. There lack studies examining mandatory CTAs from the perspective of visitors, let alone from the perspective of Chinese tourists who also use mandatory CTAs. Secondly, most research on CTAs is qualitative, and there is a lack of quantitative empirical research.

## 3 Research methods

### 3.1 Research design

First, we invited research experts, tourism workers in China, and CASM assistants to install and register Mysejahtera to submit their feedback. Questionnaires were initially designed in English, according to our feedback. The second English questionnaire was applied to ten Chinese international tour leaders and ten international students in Malaysia as a pilot test. After consulting the respondents' feedback, the questionnaires were translated into Chinese to proceed with further research concerning delivering the actual message to the respondents. We followed the translation playbook (Eremenco et al., 2005) to translate the script into Chinese and surveyed ten experienced Chinese international tourists as a pilot test in the mother tongue. The final questionnaires were modified according to the Chinese version of the pilot test on ten tourists; the result shows that the overall test only costs 10 min to complete.

The questionnaire comprises three parts; the first part is demographic variables analysis, including (1) age; (2) gender; (3) educational background; (4) marital status (5) region belonging; (6)whether installed CTA; and (7)app consulting frequency. The second part adopted the Likert scale survey to put forward questions concerning Mysejahtera to find answers for the research objects. The rejection is scaled one point in total, and the approval is made up of 5 points; the medium response goes according to this scaling approach. Meanwhile, we promised to keep attendant responses confidential to only apply for research purposes. The quantitative chart intended for research model structure use is modified according to the former version below (Table 1). In the third part, no quantitative charts are applied; the respondents express their subjective views regarding needed improvements in the Mysejahtera. The third part also applied the Likert scale approach for research; the higher the score assigned, the bigger the room gets for improvement.

**Table 1:** Measurement scales.

Stage	Variable	Subject matter	Literature sources
Part II	1. Privacy and human rights	1.1 I do not feel that collecting location information is an invasion of privacy	(Ameen et al., 2022; O'Callaghan et al., 2020)
		1.2 Mandatory use of MYSEJAHTERA does not violate human rights	
	2. Language issues	2.1 Registration language is not Chinese, feel uncomfortable	(Ying et al., 2018; von Wyl et al., 2021)
		2.2 Confused and upset by using the interface in English	
		2.3 I feel confused and helpless when answering questions during use and when submitting documents about vaccination, etc.	
		2.4 While using MYSEJAHTERA in both English and Malay, I feel uneasy and confused	
	3. registration issues other than language	3.1 I am bothered when I can't use a Chinese mobile number when registering	(Bender et al., 2014; Son et al., 2020)
		3.2 I do not know how to fill in a permanent address as a visitor	
	4. Concerning the mutual recognition of vaccines in the MYSEJAHTERA APP	4.1My vaccination record is only in Chinese only and there is no English version, so I am therefore confused.	(Bender et al., 2014; Bell et al., 2020)
		4.2 It takes 4–6 weeks to review vaccination materials, which is too long	
	5. On support and trust	5.1 Confidence in the Government of Malaysia	(Moore et al., 2021; Ameen et al., 2022; Lwin et al., 2007)
		5.2 Trust in science	
		5.3 I think an app like MYSEJAHTERA would help the government to manage	
		5.4 I think an app like MYSEJAHTERA helps with outbreak prevention and control	
Part III	Overall, for visitors how much room do you think there is for improvement in this software?		(Dean and Sharfman, 1993)

### 3.2 Sampling strategy

Chinese visitors are not allowed to travel to Malaysia as the country has mapped Malaysia as a high-risk area for COVID-19. Therefore, the experiment group turned to CITS (China International Travel Service) to recruit experienced Chinese tourists with overseas travel histories as respondents. 443 respondents completed the questionnaires, while 47 did not finish them, with a valid feedback rate of 90.41%. The control group appointed China Student Association Malaysia (CASM) to invite Chinese international students in Malaysia to fill out the questionnaires. Among the 532 overall participants, 504 completed the form, while 57 were not finishing it, which made up to 477 valid answer sheets, with a feedback rate of 84.02%.

Prior to the survey, we conducted a screening of potential respondents. Respondents in the experiment group were screened to ensure they had an international tourism experience. The control group attendees also confirmed that they are not Chinese locals in Malaysia. The research lasted from 28 October 2021 to 25 December 2021. Both the research was conducted for both groups on WeChat, a widely used instant messaging app in China. All members completed the questionnaires independently and received two dollars of bonus.

### 3.3 Estimation techniques

In the first part, except for demographical statistics, the research also included variables related to the questions, such as "whether the respondent has installed the CTAs in his hometown” or users' adoption frequency on the app. We researched two groups' variable disparity by concluding and categorizing the percentage of the comparable data and applying the chi-squared test. The sample size is measured with the minimum expected count, based on whether Pearson's chi-squared test or fisher's exact test should be used. The Cronbach alpha test is carried out in the second part of the scale to measure its effectiveness and reliability. Before analyzing the different groups' experience on Mysejahtera, the research investigated the normal distribution and accordingly chose to use independent sample T testing or Mann-Whitney U testing jointly with the median unit referral chart and proceeded with further analysis of the disparity between the two groups. Kolmogorov-Smirnov and Shapiro-Wilk are used for normality test. In the third part, we adopted used Durbin-Watson (DW) figures to determine if sample independence between the experimental and control groups is good, and VIFs between variables were also tested to ensure that there is no co-linearity between variables. The multivariate linear regression analysis was applied to decide which variables in the experiment group affected respondents' evaluation of potentially needed improvements in Mysejahtera. The data analysis proceeded with IBM SPSS 22.0. P ≤ 05 is regarded as statistically significant.

## 4 Results

### 4.1 Demographics

Descriptive analyses of the samples are conducted first from the following aspects: gender, age, educational background, marital status, area distribution, and downloading and use of digital health apps, among others. Chi-squared tests are also applied to analyze the discrepancy between different kinds. Table 2 provides the demographic characteristics of 890 people in two groups.

**Table 2:** Characteristics of demographic variables of the surveyed samples (n = 890).

	China Experimental Group	Malaysia Control Group		
	(n = 443)	(n = 447)		
Characteristic	N (%)	N (%)	χ^2^	$\sqrt{P-value}$
**Gender**			138.806	<.001
Female	327 (73.8)	154 (34.5)		
Male	116 (26.2)	293 (65.5)		
**Age**			571.181	<.001
18–20 years old	40 (9.0)	0 (0.0)		
20–25 years old	51 (11.5)	233 (52.1)		
26–30 years old	46 (10.4)	214 (47.9)		
31–40 years old	138 (31.2)	0 (0.0)		
41–50 years old	125 (28.2)	0 (0.0)		
Above 50 years old	43 (9.7)	0 (0.0)		
**Educational Background**			2.457	0.293
Under 12 Years of Education	95 (21.4)	93 (20.8)		
Bachelor's Degree	273 (61.6)	260 (58.2)		
Master's Degree and Above	75 (16.9)	94 (21.0)		
**Marital Status**			321.223	<.001
Married or Shacking Up	342 (77.2)	77 (17.2)		
Single or Divorced	101 (22.8)	370 (82.8)		
**Area Distribution**			37.766	<.001
Northeast China	41 (9.3)	44 (9.8)		
North China	35 (7.9)	49 (11.0)		
Central of China	78 (17.6)	103 (23.0)		
East China	135 (30.5)	61 (13.6)		
South China	89 (20.1)	106 (23.7)		
Northwest China	36 (8.1)	45 (10.1)		
Southwest China	29 (6.5)	39 (8.7)		
**Do they download health &CTA?**			0.122	0.727
Yes	383 (86.5)	390 (87.2)		
No	60 (13.5)	57 (12.8)		
**How often do they use health &CTA?**			92.462	<.001
Once or more than once per day	387 (87.4)	264 (59.1)		
Once or several days per week	33 (7.4)	86 (19.2)		
Never or less than once per week	23 (5.2)	97 (21.7)		

Without special notes, group differences should be assessed with $x^{2}$.

a: minimum expected count: 222.5.

As the minimum expected count is 222.5 and the sample size is 890 (much larger than 40), Pearson's chi-squared test is considered appropriate. According to the test results, there are apparent differences between experimental and control groups in gender, age, marital status, area distribution, and frequency of using health & CTA. Concerning gender, females in the experimental group take up a larger proportion of 73.8% (327/443), whereas 65.5% (293/447) of the respondents in the control group are males; χ2 = 138.806, P < 0.001. The experimental group shows a scatter of age distribution, and 31.2% (138/443) of the respondents are between 31 and 40 years old, but the respondents in the control group are relatively young on the whole, and 52.1% (233/447) of them are from 20 to 25 years old; χ2 = 571.181, P < 0.001. In terms of marital status, the two groups contrast sharply. In the experimental group, 77.2% (342/443) of the respondents are either married or shacking up with somebody, while 82.8% (370/447) of the respondents in the control group are either single or divorced; χ2 = 321.223, P < 0.001. In light of the areas where the respondents come from, 30.5% (35/443) of them in the experimental group are from East China, and 23.7% (106/447) of them in the control group come from South China; χ2 = 37.766, P < 0.001. Moreover, the proportion of using the CTA at least once per day in the experimental group reaches up to 87.4% (387/443), while that in the control group is 59.1% (264/447); χ2 = 92.462; P < 0.001.

This research discovers that there is no clear gap between the two groups concerning the educational backgrounds of the respondents, most of whom have obtained bachelor's degrees (experimental group: n = 273, 61.6%, control group: n = 260, 58.2%; χ2 = 2.457, P = 0.293). A similar result can also be gained when the respondents are asked whether they have downloaded CTA, and most of them have downloaded the app in the two groups (experimental group: n = 383, 86.5%, control group: n = 390, 87.2%; χ2 = 0.122, P = 0.727).

### 4.2 Difference feelings of using Mysejahtera among different groups

As for the scale questions in the second part, firstly, the validity and reliability of the testing chart applied Cronbach α testing approach. The analysis result manifests that the alpha ecoefficiency for privacy and human rights is 0.567, for language barrier is 0.877, for registration and signing is 0.879, for vaccine mutual recognition is 0.674, and for trust and support is 0.636. In general, alpha ecoefficiency above 0.6 guarantees the reliability of the questionnaire chart (Nunnally and Bernstein, 1994). Regarding social science research, such as privacy and human rights, the marginal value for its alpha ecoefficiency is 0.55 (Ziegel, 1995). Therefore, in conclusion, the evaluation factors applied in the study are reasonably organized for further assessment. Then we test the normality of the variables with the methods of the Kolmogorov-Smirnov test and Shapiro-Wilk test. Specific results are shown in Table 3. However, the data in this part fail to meet the normality distribution criteria, so the Mann-Whitney U test is used to conduct data differentiation analysis. Compared with the student's test, the Mann-Whitney U test is less likely to give falsely significant results (Siegel and John Castellan, 2000) and lose statistical power (Gibbons and Chakraborti, 1991).

**Table 3:** Summary table for the normality test (n = 890).

Variable	Kolmogorov-Smirnova		Shapiro-Wilk	
	Statistic	Sig.	Statistic	Sig.
Privacy & Human Rights				
I don't think collection of location information is an invasion of privacy.	0.458	<.001	0.570	<.001
I don't think mandatoryuse of Mysejahtera is an invasion of privacy.	0.470	<.001	0.546	<.001
Language Difficulties				
I feel uncomfortable without Chinese translation when registering.	0.296	<.001	0.763	<.001
I feel disconcerted with English interface.	0.294	<.001	0.762	<.001
I feel disconcerted to answer questions and submit vaccine-related materials in English.	0.297	<.001	0.753	<.001
I feel disconcerted with both English and Malay as the official languages of MYSEJAHTERA	0.523	<.001	0.383	<.001
Registration Difficulties Other Than Language				
I feel perplexed that Chinese mobile phone number cannot be used to register.	0.226	<.001	0.853	<.001
As a visitor, I have no idea how to write permanent address.	0.242	<.001	0.825	<.001
On the Issues of Vaccine Registration of Mutual Recognition in MYSEJAHTERA				
I feel confused that vaccination is only reflected in Chinese health code and without English version.	0.528	<.001	0.357	<.001
4–6 weeks are too long for reviewing vaccine-receiving materials.	0.521	<.001	0.394	<.001
About Support & Trust				
Trust in Malaysian government.	0.518	<.001	0.405	<.001
Trust in science.	0.520	<.001	0.398	<.001
I think such apps as Mysejahtera will help government management.	0.521	<.001	0.392	<.001
I think such apps as Mysejahtera will help pandemic control.	0.519	<.001	0.402	<.001
Improvements				
Generally, how much room do you think does this app have to improve from a visitor's perspective?	0.246	<.001	0.872	<.001

As is shown in Table 4, there is no discrepancy between the two variables concerning privacy and human rights (the two medians are both 5, P = 0.231 and 0.719). In other words, the respondents of the two groups neither consider location information collection nor mandatory use of Mysejahtera as an invasion of privacy. Similarly, the medians of the four variables about support and trust in both experimental and control groups represent the respondents' willingness to give firm belief in local government and science, as well as in the positive role that Mysejahtera itself will play in helping prevent and control Covid-19, and government management of the pandemic (the medians in the two groups are 5, P = 0.880 and P = 0.218 and P = 0.727 and P = 0.880).

**Table 4:** Summary sheet of Mann-Whitney U test (n = 890).

Variable	Average	Median	Standard Deviation	Average Rank	Z value	P value
I don't think collection of location information is an invasion of privacy.					−1.197	0.231
Experimental Group	4.700	5.000	0.488	437.530		
Control Group	4.740	5.000	0.471	453.400		
I don't think mandatory use of Mysejahtera is an invasion of privacy.					−0.360	0.719
Experimental Group	4.740	5.000	0.474	447.840		
Control Group	4.730	5.000	0.473	443.180		
I feel uncomfortable without Chinese translation when registering.					−27.833	<.001
Experimental Group	4.900	5.000	0.302	669.000		
Control Group	1.990	2.000	0.521	224.000		
I feel disconcerted with English interface.					−27.827	<.001
Experimental Group	4.890	5.000	0.308	669.000		
Control Group	2.050	2.000	0.514	224.000		
I feel disconcerted to answer questions and submit vaccine-related materials in English					−27.977	<.001
Experimental Group	4.900	5.000	0.302	669.000		
Control Group	1.970	2.000	0.477	224.000		
I feel disconcerted with both English and Malay as the official languages of MYSEJAHTERA					−1.892	0.059
Experimental Group	4.900	5.000	0.302	454.800		
Control Group	4.860	5.000	0.351	436.290		
I feel perplexed that Chinese mobile phone number cannot be used to register.					−24.843	<.001
Experimental Group	4.710	5.000	0.486	651.130		
Control Group	2.980	3.000	0.744	241.710		
As a visitor, I have no idea how to write permanent address.					−26.610	<.001
Experimental Group	4.740	5.000	0.481	666.420		
Control Group	2.040	2.000	0.739	226.560		
I feel confused that vaccination is only reflected in Chinese health code and without English version.					−0.935	0.350
Experimental Group	4.880	5.000	0.328	450.260		
Control Group	4.860	5.000	0.351	440.790		
4–6 weeks are too long for reviewing vaccine-receiving materials.					−0.247	0.805
Experimental Group	4.860	5.000	0.352	444.210		
Control Group	4.860	5.000	0.346	446.780		
Trust in Malaysian government					−0.151	0.880
Experimental Group	4.860	5.000	0.345	444.720		
Control Group	4.870	5.000	0.341	446.270		
Trust in science					−1.232	0.218
Experimental Group	4.880	5.000	0.329	451.700		
Control Group	4.860	5.000	0.351	439.360		
I think such apps as Mysejahtera will help government management.					−0.350	0.727
Experimental Group	4.880	5.000	0.330	447.250		
Control Group	4.870	5.000	0.339	443.760		
I think such apps as Mysejahtera will help pandemic control.					−0.151	0.880
Experimental Group	4.860	5.000	0.343	444.730		
Control Group	4.870	5.000	0.339	446.260		
Generally, how much room do you think does this app have to improve from a visitor's perspective?					−0.028	0.977
Experimental Group	2.980	3.000	0.822	445.270		
Control Group	2.990	3.000	0.803	445.720		

With the Mann-Whitney U test, it is discovered that the discrepancies between the two groups mainly lie in two factors: one is that Mysejahtera has only an English version, and the other is the inconvenience in registration and login processes. The medians of the variables in the experimental group are 5, and those in the control group are 2 or 3, thus making P < 0.001. This result indicates that the experimental group is far more disturbed in terms of several variables than the control group.

The results reveal some common problems regarding both English and Malay as official languages (P = 0.059) and mutual recognition of vaccines (P = 0.350 and P = 0.805) in Mysejahtera. There is no obvious difference among the three variables in the two groups, but instead, two medians of the three are 5, which explains the common ground of the three variables. However, the result of the independent scale questions in part 3 reveals that all the respondents believe Mysejahtera has improvement space (P = 0.977, the medians are 3). The experimental group is more easily disturbed, but the existence of common problems does not necessarily mean the variables will influence the use of Mysejahtera, nor the improvement of Mysejahtera Therefore, further analysis of relevant variables will be carried out with the method of multiple linear regression.

### 4.3 Influences of use situation on how to improve

Finally, with the method of multiple linear regression, the discussion of whether use situation has obvious influences upon how to improve in different groups will be conducted. The Durbin-Watson (DW) figures from the two groups show the samples are fairly independent (the experimental group: 2.094, the control group: 1.956). All the VIFs among variables are under 2, which means the calculation results are accurately reliable and there exists no multicollinearity among variables. As for re-squared analysis, the results reveal that *R*^2^*R*^2^ of the experimental group is 0.347 and that of the control group is 0.489. On the whole, according to the research of Knofczynski and Mundfrom (2007) and the comparison results of *R*^2^ in both experimental and control groups, the sample size of this multiple linear regression analysis reaches good prediction level.

Table 5 shows the results of the linear regression analysis. The results deduced with regression analysis are the same as those obtained through the Mann-Whitney U test, which means that such variables have nothing to do with improvement space. They are: “I don't think collection of location information is an invasion of privacy”. (P = 0.800, 95% CI: [−0.120,0.155]); “I do not think mandatory use of Mysejahtera is an invasion of privacy”. (P = 0.071 95% CI: [−0.011, 0.273]), trust in Malaysian government (P = 0.434, 95% CI:[−0.120, 0.278]), and similar apps like Mysejahtera are believed to help government manage pandemic matters, as well as help, prevent and control Covid-19 (P = 0.094, 95% CI: [−0.030,0.387]; P = 0.628, 95% CI: [−0.261, 0.158]). The three variables considered to have joint problems through the Mann-Whitney U test are not verified by multiple linear regression results (P = 0.052, 95% CI: [−0.002, 0.446]; P = 0.090, 95% CI: [−0.029, 0.408]; P = 0.677, 95% CI: [−0.245, 0.159]), so the three variable are thought to be unrelated to improvement.

**Table 5:** Regression analysis of influences of use situation on how to improve in different groups (n = 890).

Group	Variable	b	S.E.	Beta	t	Sig.	95% Confidence Interval
China	Constant	−12.410	1.253		−9.907∗∗∗	<.001	[−14.872, −9.948]
	I don't think collection of location information is an invasion of privacy.	0.018	0.070	0.010	0.253	0.800	[−0.120, 0.155]
	I don't think mandatory use of Mysejahtera is an invasion of privacy.	0.131	0.072	0.075	1.809	0.071	[−0.011, 0.273]
	I feel uncomfortable without Chinese translation when registering.	0.517	0.110	0.190	4.694∗∗∗	<.001	[0.300, 0.733]
	I feel disconcerted with English interface.	0.495	0.115	0.186	4.312∗∗∗	<.001	[0.269, 0.721]
	I feel disconcerted to answer questions and submit vaccine−related materials in English.	0.281	0.114	0.103	2.461∗	0.014	[0.057, 0.506]
	I feel disconcerted with both English and Malay as the official languages of MYSEJAHTERA	0.222	0.114	0.082	1.949	0.052	[−0.002, 0.446]
	I feel perplexed that Chinese mobile phone number cannot be used to register.	0.384	0.070	0.227	5.509∗∗∗	<.001	[0.247, 0.522]
	As a visitor, I have no idea how to write permanent address.	0.547	0.071	0.320	7.702∗∗∗	<.001	[0.407, 0.687]
	I feel confused that vaccination is only reflected in Chinese health code and without English version.	0.190	0.111	0.075	1.702∗∗∗	0.090	[−0.029, 0.408]
	4–6 weeks are too long for reviewing vaccine−receiving materials.	−0.043	0.103	−0.018	−0.417	0.677	[−0.245, 0.159]
	Trust in Malaysian government	0.079	0.101	0.033	0.784	0.434	[−0.120, 0.278]
	Trust in science	0.235	0.106	0.094	2.215∗	0.027	[0.026, 0.443]
	I think such apps as Mysejahtera will help government management.	0.178	0.106	0.072	1.680	0.094	[−0.030, 0.387]
	I think such apps as Mysejahtera will help pandemic control.	−0.052	0.107	−0.022	−0.485	0.628	[−0.261, 0.158]
Malaysia	Constant	−3.465	0.874		−3.966∗∗∗	<.001	[−5.182, −1.748]
	I don't think collection of location information is an invasion of privacy.	−0.061	0.066	−0.036	−0.924	0.356	[−0.192, 0.069]
	I don't think mandatory use of Mysejahtera is an invasion of privacy.	0.185	0.066	0.109	2.798∗∗	0.005	[0.055, 0.314]
	I feel uncomfortable without Chinese translation when registering.	0.296	0.058	0.192	5.095∗∗∗	<.001	[0.182, 0.411]
	I feel disconcerted with English interface.	0.338	0.061	0.217	5.552∗∗∗	<.001	[0.219, 0.458]
	I feel disconcerted to answer questions and submit vaccine−related materials in English.	0.400	0.064	0.238	6.268∗∗∗	<.001	[0.274, 0.525]
	I feel disconcerted with both English and Malay as the official languages of MYSEJAHTERA	0.040	0.084	0.018	0.480	0.632	[−0.125, 0.206]
	I feel perplexed that Chinese mobile phone number cannot be used to register.	0.227	0.040	0.210	5.662∗∗∗	<.001	[0.148, 0.306]
	As a visitor, I have no idea how to write permanent address.	0.365	0.040	0.336	9.125∗∗∗	<.001	[0.286, 0.444]
	I feel confused that vaccination is only reflected in Chinese health code and without English version.	0.195	0.090	0.085	2.169∗	0.031	[0.018, 0.372]
	4–6 weeks are too long for reviewing vaccine−receiving materials.	−0.020	0.090	−0.009	−0.224	0.822	[−0.198, 0.158]
	Trust in Malaysian government	−0.009	0.088	−0.004	−0.103	0.918	[−0.183, 0.165]
	Trust in science	0.160	0.088	0.070	1.813	0.071	[−0.013, 0.334]
	I think such apps as Mysejahtera will help government management.	0.101	0.090	0.043	1.126	0.261	[−0.075, 0.278]
	I think such apps as Mysejahtera will help pandemic control.	0.022	0.090	0.009	0.244	0.807	[−0.154, 0.198]

∗P < 0.05, ∗∗P < 0.01, ∗∗∗P < 0.001.

The multiple linear regression analyzed the following factors “I feel uncomfortable without Chinese translation when registering.”; “I feel disconcerted with English interface.”; “I feel disconcerted to answer questions and submit vaccine-related materials in English.” (P < .001, 95% CI: [0.300, 0.733]; P < .001, 95% CI: [0.269, 0.721]; P = 0.014, 95% CI: [0.057, 0.506]); “I feel perplexed that Chinese mobile phone number cannot be used to register.” and “As a visitor, I have no idea how to write permanent address.” (P < .001, 95% CI: [0.247, 0.522]; P < .001, 95% CI: [0.407, 0.687]). Improving the above five areas would significantly increase visitors' satisfaction with Mysejahtera. In addition to this, the linear regression results for the variable "Trust in science” (p < 0.027, 95% CI: [0.026,0.443]) in the experimental group showed that there is still room for improvement in this item, thus negating the inference that this item is "not a problem” as revealed by the Mann Whitney U test.

## 5 Discussions & conclusion

### 5.1 Key findings

This research aims to collect deficiencies in the Mysejahtera and conclude vital factors that might contribute to reviving international tourism in Malaysia. The research was conducted by analyzing the sentiment of the Chinese tourists regarding the Malaysian CTA, Mysejahtera, which is unprecedented as it is the first research that analyzed tourists' sentiment towards the CTA from a foreign country. The biggest issue the research spot on is that the studied tourists face problems such as language barrier and registration difficulties while using the Malaysian CTA. The research suggests that addressing these problems might revive international tourism in Malaysia, especially the Chinese tourists. Specifically, the main findings of this paper are as follows.

#### 5.1.1 Mysejahtera still has room for improvement

This study yields various exciting findings. While previous research has argued that Chinese visitors do not require special care in terms of language (Jin et al., 2016), our study argues that language issues are the first and foremost problem for Chinese visitors when using Mysejahtera. Although some research suggested that Chinese visitors have difficulties expressing themselves in English (Bolton and Graddol, 2012), we found that the Mysejahtera English application interface was not user-friendly for Chinese visitors, and most Chinese respondents found this to be a problem for them to use. In other words, the results of our current survey suggest that Chinese visitors also have some barriers to reading English. Previous studies have shown that Chinese visitors can experience communication difficulties due to language barriers, which can affect travel (Choi et al., 2008), and our study also confirms the confusion and discomfort in using Mysejahtera due to the absence of Chinese.

The research conducted by Melubo and Kisasembe (2020) in Tanzania and Zhang and Schmude in German (2021) pointed out that Chinese tourists need Chinese language assistance in a foreign country and do not necessarily need local language assistance. Our research result does not approve the claim that unfamiliarity with the Malay language prohibits users' ability to use Mysejahtera. Thus, Chinese language services should be prioritized in hosting countries' agendas to attract more Chinese tourists.

According to the conclusion made by this research, Mysejahtera might improve its functions regarding registration and signing. Our findings show that Chinese visitors find it troublesome as they cannot register the platform with China's mobile phone number. Our research supports the findings of Chen et al. (2018) in China that young Chinese people are more likely to use smartphones than computers. By the end of 2020, the number of mobile phone users in China had reached 1.6 billion. It exceeded the country's total population (Weissberger, 2020), with the majority of these users using smartphones (Zheng et al., 2021) compared to 1.01 billion computer internet users in the same period (CGTN, 2021b). In other words, smartphones are more prevalent than computers for Chinese visitors, and mobile phones for registration are more relevant to Chinese visitors. Moreover, Mysejahtera also requires users to fill in Malaysian home addresses, making the registration more confusing to Chinese tourists. Evidently, visitors usually have a temporary address only, and it is not logical for Mysejahtera to ask for a permanent address. In this regard, Mysejahtera has not been designed with the visitor's perception in mind. Those inconsiderate functions undermine the Chinese tourists' positive sentiments towards Malaysia, and it is harmful to reviving Malaysia's international tourism industry.

The linear regression test in our study also rejects the inference derived from the Mann-Whitney U test, namely, the variable “trust in science” is “not a problem,” and our findings suggest that there is still room for improvement in this item. This result is in line with the findings of Plohl and Musil (2020) in France that the more trust the respondents show for science, the less they are expected improvements in Mysejahtera. Possible reasons for CTA users' skepticism about science are that the population is influenced by untruthful information on social media or is under the spell of right-leaning media and populism (Pennycook et al., 2020; Motta et al., 2020; Eberl et al., 2021).

#### 5.1.2 The CTA embraced respondents’ strong acknowledgments

According to the research, 86.5% of the respondents (n = 383/443) in the experiment group have installed a Chinese health code application, higher than the 64.3% obtained by the research made by Liang (2020) and Tencent (2020). Besides, the result of the article shows that the two group respondents are mainly made up of young generations, with the majority of them possessing bachelor's degrees; however, those respondents are the people who widely apply the CTAs. The result aligns with the idea of Chen et al. (2020), which proposes that the young generations are good at technological acknowledgment. Educated people tend to reach out to modern media and possess a high cognitive ability to use CTAs (Chan et al., 2021). The research conducted by Harith et al., in 2021 claims that the CTAs promoted in Malaysia are identical to the health code implemented in China, regarding both applying data tracking and requiring users to scan QR codes while entering public spaces (Harith et al., 2021). The research article suggests that the respondents from two groups claim that they use CTAs at least once or more per day. This argument indirectly supported the above conclusions.

Users' supports are necessary for the effectiveness of mandatory CTAs, and the survey results show that the respondents support using this type of CTA. Previous studies have pointed out that mandatory CTA violates people's privacy and human rights (BASU, 2020). However, the two groups in our study denounce the claim that the CTA violates privacy and do not see CTA as a human rights violation. The results of this study also confirm the findings of Huang (2020) that Chinese visitors differ from other international travelers due to political and cultural reasons. Previous surveys on CTA use have mainly focused on voluntary CTA users, using qualitative research methods such as survey interviews to understand their perceptions of use, privacy protection, and room for improvement (John Leon Singh et al., 2020; Chan and Saqib, 2021; Rowe, 2020). This empirical study examines respondents' trust in the Malaysian government through quantitative analysis, obtaining findings that are consistent with the results of Wu et al. (2021) that people are satisfied with how the government controls the COVID-19 pandemic and thus showed a high level of trust in the government. According to the study, the respondents claim that CTA helped harness the pandemic, and Mysejahtera showed a magnificent result. As stated in the study by Villius Zetterholm et al. (2021), the effectiveness of CTAs depends largely on public acceptance and use. According to our study, respondents were willing to use Mysejahtera, with 87.4% of the experimental group using the CTA at least once daily. The results indicate that the CTA has a high frequency of use and that pandemic control effectiveness in China and Malaysia is closely related to the cooperation of users.

Our study found that the issues involved in Malaysia's registration of certificates for vaccinations administered in China are not relevant to whether there is room for improvement in Mysejahtera. This finding assuages the concerns raised when designing the questionnaire and differs from the results obtained through the Mann-Whitney U Test.

### 5.2 Theoretical contribution& practical implication

Compared to previous studies, this paper investigates statistically whether there is room for improvement in using the mandatory CTA Mysejahtera from the perspective of Chinese visitors. The followings are the main theoretical contributions:

(1) The most significant one is that this paper innovates the research perspective of CTA. Most previous studies have been conducted on the possible privacy issues and acceptance of the voluntary CTA from the residents' perspective. These studies mainly discuss the technical features of the apps themselves, neglecting the study of whether there is room for improvement in the use of Mysejahtera by tourists. This paper examines the issues from the perspective of Chinese visitors, who also use mandatory CTA and are essential to Malaysia's international tourism industry. Our study is significant in that it fills this research gap.
(2) Previous studies on CTAs have mostly been qualitative. In this paper, we use a quantitative approach to study the language barrier, inconvenience in registration, the distrust of science from the perspective of international visitors, and many other issues to be improved. In this regard, our study provides additional options for research methodology on such issues.

Based on the study's findings, this paper offers the following practical insights for national functionaries using mandatory CTA, including Malaysia and China.

(1) Language barrier restricts the use of Mysejahtera. As many Chinese visitors cannot read English well, relevant authorities can develop a Chinese version of app. Secondly, Chinese mobile phone numbers should be enabled for registration. Thirdly, visitors should be allowed to fill in the temporary addresses, including hotels and B&Bs (Bed and Breakfast), instead of a permanent one. The experience of Chinese visitors will be significantly enhanced by improving the above problems.
(2) Government departments can invest more in science and promote the app by inviting influential celebrities, stars, and famous personalities for endorsement, which will increase its recognition and influence. Also, more efforts are needed to invest more in epidemic prevention. Mysejahtera can also be used as a promotion platform for pandemic prevention posts and videos to enhance people's awareness.

### 5.3 Limitations and vision for the future work

A comparison of the demographic aspects of this study with the data published by the National Bureau of Statistics of China reveals that the cross-sectional design of the sample for this survey hinders the examination of the cause-and-effect relations. Our research sample is narrowed to the oversea tourism experienced communities compared to the significant nationwide research. Notably, the bachelor's degree attendees comprise 60% of whole respondents (experiment group: 61.6%, control group: 58.2%), and the figure is much higher than the percentage of 15% (China National Bureau of Statistics, 2012), which was stated in official data. Although we chose samples from ordinary crowds, respondents were still not representative of the general nationwide population. From the perspective of the age structure of respondents, this research neglected older generations. However, according to the study "tourism behavior of older generations” made by Ctrip (2019), an online Chinese tourism platform, 65% of the respondents of elder age claim that they at least travel more than three times a year. In future research, older generations should be involved.

Knowing the demand of the older generations in COVID-19 is also the primary concern in developing apps regarding the health and mental well-being of the older generation. Unsurprisingly, the Chinese elder generations will be much more confused facing the English interface of Mysejahtera than the younger respondents (Wang and Jia, 2020), so if the research has also involved the older generation, the result might be different.

In the COVID-19 pandemic, Chinese tourism to other countries was halted. Although the experiment group members have international tourism experience and once installed Mysejahtera, they still have some problems using the app. Thus, after China opens the border for tourists to go out, we may need to conduct the research again to collect feedback from tourists who have been to Malaysia.

This research concludes that the questions asked by the Mysejahtera in recognizing the Chinese domestically vaccinated tourists are not associated with the respondents' view over” if there is still room for improvement in Mysejahtera.” However, controversially, the median number for relative variables is 5, which suggests abnormality and needs further analysis.

Another drawback of this study lies in the sampling frame. The research and the CTA attendees came from a country where tracing people for pandemic prevention is compulsory. If the attendees are someone who belongs to a country in which the CTA is not compulsory, then the research statement will be made differently. In fact, except for Malaysia, the countries which turned to coercive methods to popularize CTAs are only made up of a few, such as China, Singapore, India, and Korea (Rowe et al., 2020). Most counties allow citizens to install the app according to their willingness. In the future, we expect to expand the research target and analyze the tourists from countries where CTAs are not compulsory.

## 6 Conclusions

This quantitative empirical study investigates the experience of using the mandatory CTA Mysejahtera from the perspective of Chinese visitors. This study conveys two messages. First, language barriers are the most critical factor preventing Chinese visitors from Nota efficiently. Not being able to use Chinese mobile phone numbers for registration causes unnecessary distress to Chinese visitors and significantly impacts the routine use of Mysejahtera. Second, visitors' trust in science positively correlates with the broader application of mandatory CTAs such as Mysejahtera. Service providers are advised to allow Chinese visitors to register with a mobile phone number of foreign countries and to use a temporary address instead of a permanent address. A Chinese version of Mysejahtera should be developed and utilized to promote the importance of vaccination to increase users' trust in science.

## Declarations

### Author contribution statement

Xiaocong Jiang: Conceived and designed the experiments; Performed the experiments; Analyzed and interpreted the data; Wrote the paper.

Ahmad Edwin Mohamed: Conceived and designed the experiments; Contributed reagents, materials, analysis tools or data.

### Funding statement

This work was supported by the Philosophy and Social Science Research Project of Jiangsu Universities. [ No. 2021SJA1476].

### Data availability statement

Data will be made available on request.

### Declaration of interest's statement

The authors declare no conflict of interest.

### Additional information

No additional information is available for this paper.

## References

[bib1] John Leon Singh H., Couch D., Yap K. (2020). Mobile health apps that help with COVID-19 management: scoping review. JMIR Nurs..

[bib2] Abd-Alrazaq A., Alhuwail D., Househ M., Hamdi M., Shah Z. (2020). Top concerns of Tweeters during the COVID-19 pandemic: infoveillance study. J. Med. Internet Res..

[bib3] Abdali T.-A.N., Hassan R., Mohd Aman A.H. (2021). A new Feature in Mysejahtera Application to Monitoring the Spread of COVID-19 using fog computing. IEEE Xplore.

[bib4] Abeler J., Bäcker M., Buermeyer U., Zillessen H. (2020). Covid-19 contact tracing and data protection can go together (Preprint). JMIR MHealth UHealth.

[bib5] Aji Z.M., Mohd Salleh N.S., Zakaria N.H., Mohd Khalid A.H. (2021). 2021 7th International Conference on Research and Innovation in Information Systems (ICRIIS).

[bib6] Akinbi A., Forshaw M., Blinkhorn V. (2021). Contact tracing apps for the COVID-19 pandemic: a systematic literature review of challenges and future directions for neo-liberal societies. Health Inf. Sci. Syst..

[bib7] Altmann S., Milsom L., Zillessen H., Blasone R., Gerdon F., Bach R., Kreuter F., Nosenzo D., Toussaert S., Abeler J. (2020). Acceptability of app-based contact tracing for COVID-19: cross-country survey study. JMIR MHealth UHealth.

[bib8] Ameen N., Hosany S., Paul J. (2022). The personalisation-privacy paradox: consumer interaction with smart technologies and shopping mall loyalty. Comput. Hum. Behav..

[bib9] Amin E.P.P., Ker P.J., Leong Y.S., Ismail A., Gurbachan Singh B.S. (2020). Malaysian multiphasic health screening caravan (MyMHSCar) – an automatic device for health screening and attendance system. Journal of Energy & Environment.

[bib11] Ateljevic I., Doorne S. (2000). “Staying within the fence”: lifestyle Entrepreneurship in tourism. J. Sustain. Tourism.

[bib12] Basu S. (2020). Effective contact tracing for COVID-19 using mobile phones: an ethical analysis of the mandatory use of the Aarogya Setu application in India. Camb. Q. Healthc. Ethics.

[bib13] Bell S., Clarke R., Mounier-Jack S., Walker J.L., Paterson P. (2020). Parents’ and guardians’ views on the acceptability of a future COVID-19 vaccine: a multi-methods study in England. Vaccine.

[bib14] Bender M.S., Choi J., Arai S., Paul S.M., Gonzalez P., Fukuoka Y. (2014). Digital technology ownership, usage, and factors predicting downloading health apps among caucasian, Filipino, Korean, and latino Americans: the digital link to health survey. JMIR MHealth UHealth.

[bib15] Bibbiani T. (2020). How do contact tracing apps work?. Top Mobile App Dev..

[bib16] Bolton K., Graddol D. (2012). English in China today. Engl. Today.

[bib17] CGTN (2021). China’s Ruili launches second citywide nucleic acid testing. News.cgtn.com. https://news.cgtn.com/news/2021-04-07/China-s-Ruili-launches-second-citywide-nucleic-acid-testing-ZgjjT2Vx4c/index.html.

[bib18] CGTN (2021). China’s netizen population hits over 1 billion. News.cgtn.com. https://news.cgtn.com/news/2021-08-27/China-s-netizen-population-hits-over-1-billion-133ONCedM5i/index.html.

[bib19] Chan E.Y., Saqib N.U. (2021). Privacy concerns can explain unwillingness to download and use contact tracing apps when COVID-19 concerns are high. Comput. Hum. Behav..

[bib20] Chan T.-J., Wok S., Sari N.N., Muben M.A.H.A. (2021). Factors influencing the intention to use Mysejahtera application among malaysian citizens during COVID-19: a PLS-SEM analysis. J. of Applied Structural Equation Modeling.

[bib21] Chen L., Liu R., Zeng H., Xu X., Zhu R., Sharma M., Zhao Y. (2018). Predicting the time spent playing computer and mobile games among medical undergraduate students using interpersonal relations and social cognitive theory: a cross-sectional survey in chongqing, China. Int. J. Environ. Res. Publ. Health.

[bib22] Chen C.-M., Jyan H.-W., Chien S.-C., Jen H.-H., Hsu C.-Y., Lee P.-C., Lee C.-F., Yang Y.-T., Chen M.-Y., Chen L.-S., Chen H.-H., Chan C.-C. (2020). Containing COVID-19 among 627,386 persons in contact with the diamond princess cruise ship passengers who disembarked in Taiwan: big data analytics. J. Med. Internet Res..

[bib23] China National Bureau of Statistics (2012). National data. Stats.gov.cn. https://data.stats.gov.cn/easyquery.htm?cn=C01&amp;zb=A0305&amp;sj=2020.

[bib24] Chiu N.-C., Chi H., Tai Y.-L., Peng C.-C., Tseng C.-Y., Chen C.-C., Tan B.F., Lin C.-Y. (2020). Impact of wearing masks, hand hygiene, and social distancing on influenza, Enterovirus, and all-cause pneumonia during the coronavirus pandemic: retrospective national epidemiological surveillance study. J. Med. Internet Res..

[bib25] Choi T.-M., Liu S.-C., Pang K.-M., Chow P.-S. (2008). Shopping behaviors of individual tourists from the Chinese Mainland to Hong Kong. Tourism Manag..

[bib26] CodeBlue (2020). Government to legislate MySejahtera use for all business | CodeBlue. CodeBlue. https://codeblue.galencentre.org/2020/08/03/government-to-legislate-mysejahtera-use-for-all-business/.

[bib27] Ctrip (2019). Report on the travel behavior of the Elderly in China. Wappass.baidu.com. https://baijiahao.baidu.com/s?id=1646687962391413456&amp;wfr=spider&amp;for=pc.

[bib28] Dai Y.-D., Chen K.-Y., Gong X., Zhuang W.-L., Li A.-N., Huan T.-C. (2019). Developing Chinese tourist’s leisure literacy scale from the perspective of Chinese culture. Tourism Manag. Perspect..

[bib29] Dean J.W., Sharfman M.P. (1993). Procedural rationality IN the strategic decision-making process∗. J. Manag. Stud..

[bib30] Department of Statistics, Malaysia (2020). Snapshot statistics 2019 by department of statistics Malaysia official portal. https://www.dosm.gov.my/v1/index.php?r=column/cone&amp;menu_id=aG0ybDVybU9TNzdKSVR5MllKVTlQdz09.

[bib31] Eberl J.-M., Huber R.A., Greussing E. (2021). From populism to the “plandemic”: why populists believe in COVID-19 conspiracies. J. Elections, Public Opin. Parties.

[bib32] Eremenco S.L., Cella D., Arnold B.J. (2005). A comprehensive method for the translation and cross-cultural validation of health status questionnaires. Eval. Health Prof..

[bib33] Esbin M.N., Whitney O.N., Chong S., Maurer A., Darzacq X., Tjian R. (2020). Overcoming the bottleneck to widespread testing: a rapid review of nucleic acid testing approaches for COVID-19 detection. RNA.

[bib34] Fan Y. (2000). A classification of Chinese culture. Cross Cult. Manag.: Int. J..

[bib35] Fox G., Clohessy T., van der Werff L., Rosati P., Lynn T. (2021). Exploring the competing influences of privacy concerns and positive beliefs on citizen acceptance of contact tracing mobile applications. Comput. Hum. Behav..

[bib36] Gerli P., Arakpogun E.O., Elsahn Z., Olan F., Prime K.S. (2021). Beyond contact-tracing: the public value of eHealth application in a pandemic. Govern. Inf. Quart..

[bib37] Gibbons J.D., Chakraborti S. (1991). Comparisons of the mann-whitney, student’st, and AlternatetTests for means of normal distributions. J. Exp. Educ..

[bib38] Harith A.A., Muhamad N.A., Griffiths R. (2021). Digital contact tracing in combating COVID-19 pandemic in Malaysia, New Zealand and China (preprint). JMIR.

[bib39] Hinch R., Probert W., Nurtay A., Kendall M., Wymant C., Hall M., Lythgoe K., Bulas Cruz A., Zhao L., Stewart A., Ferretti L., Parker M., Meroueh A., Mathias B., Stevenson S., Montero D., Warren J., Mather N., Finkelstein A., Abeler-Dörner L. (2020). Effective configurations of a digital contact tracing app: a report to NHSX. https://cdn.theconversation.com/static_files/files/1009/Report_-_Effective_App_Configurations.pdf?1587531217.

[bib40] Howell B., Potgieter P.H. (2021). A tale of two contact-tracing apps – comparing Australia’s CovidSafe and New Zealand’s NZ Covid Tracer. Digit. Pol., Regul. Governance.

[bib41] Huang Y. (2020). China’s approach to containing coronavirus cannot be replicated. http://Www.aljazeera.com.

[bib42] Jin X., Wu L., Becken S., Ding P. (2016). How do worry, self-efficacy, and coping interact? Examining Chinese tourists to Australia. J. China Tourism Res..

[bib43] Knofczynski G.T., Mundfrom D. (2007). Sample sizes when using multiple linear regression for prediction. Educ. Psychol. Measure..

[bib44] Konidaris A., Stellatou O., Polykalas S.E., Katsoni V. (2021). Tourism and contact tracing apps in the COVID-19 Era. Cult. Tourism Smart, Globaliz., Sustain. World.

[bib45] Liang F. (2020). COVID-19 and health code: how digital platforms tackle the pandemic in China. Social Media + Society.

[bib46] Liu C., Graham R. (2021). Making sense of algorithms: relational perception of contact tracing and risk assessment during COVID-19. Big Data Soc..

[bib47] Liu Y., Huang K., Bao J., Chen K. (2019). Listen to the voices from home: an analysis of Chinese tourists’ sentiments regarding Australian destinations. Tourism Manag..

[bib48] Lucivero F., Hallowell N., Johnson S., Prainsack B., Samuel G., Sharon T. (2020). COVID-19 and contact tracing apps: ethical challenges for a social experiment on a global scale. J. Bioethical Inquiry.

[bib49] Lwin M., Wirtz J., Williams J.D. (2007). Consumer online privacy concerns and responses: a power–responsibility equilibrium perspective. J. Acad. Market. Sc..

[bib50] Lyu Y., Carroll J.M. (2022). Cultural influences on Chinese citizens’ adoption of digital contact tracing: a human infrastructure perspective. CHI Conf. Hum. Factors Comput. Syst..

[bib51] Macau Government Tourism Office (2021). Top ten visitor-source markets of Macau. https://dataplus.Macautourism.gov.mo/Publication/Report/TopTenVisitorSourceMarkets?year=2021&amp;month=12&amp;lang=E.

[bib52] Melubo K., Kisasembe R. (2020). We need Chinese tourists, but are we ready? Insights from the Tanzanian safari industry. J. China Tourism Res..

[bib53] Moore S.E., Wierenga K.L., Prince D.M., Gillani B., Mintz L.J. (2021). Disproportionate impact of the COVID-19 pandemic on perceived social support, mental health and somatic symptoms in sexual and gender minority populations. J. Homosexual..

[bib54] Motta M., Stecula D., Farhart C. (2020). How right-leaning media coverage of COVID-19 facilitated the spread of misinformation in the Early stages of the pandemic in the U.S.. Can. J. Political Sci..

[bib55] Mozur P., Zhong R., Krolik A. (2020). Coronavirus Fight, China Gives Citizens a Color Code, With Red Flags. The New York Times. https://www.nytimes.com/2020/03/01/business/china-coronavirus-surveillance.html.

[bib56] HKSAR. (n.d.). COVID-19 Thematic Website, Together, We fight the virus, Return2hk travel scheme for Hong Kong residents returning from mainland or Macau without being subject to quarantine under the compulsory quarantine of certain persons arriving at Hong Kong regulation (cap. 599C) (Return2hk scheme). Www.coronavirus.gov.hk. https://www.coronavirus.gov.hk/eng/return2hk-scheme.html.

[bib57] Nunnally J.C., Bernstein I. (1994). The assessment of reliability. Psychometr. Theory.

[bib58] Osmanlliu E., Rafie E., Bédard S., Paquette J., Gore G., Pomey M.-P. (2021). Considerations for the design and implementation of COVID-19 contact tracing apps: scoping review. JMIR MHealth UHealth.

[bib59] O’Callaghan M.E., Buckley J., Fitzgerald B., Johnson K., Laffey J., McNicholas B., Nuseibeh B., O’Keeffe D., O’Keeffe I., Razzaq A., Rekanar K., Richardson I., Simpkin A., Abedin J., Storni C., Tsvyatkova D., Walsh J., Welsh T., Glynn L. (2020). A national survey of attitudes to COVID-19 digital contact tracing in the Republic of Ireland. Irish J. Med. Sci..

[bib60] Park S., Choi G.J., Ko H. (2021). Privacy in the time of COVID-19: divergent paths for contact tracing and route-disclosure mechanisms in South Korea. IEEE Secur. Privacy.

[bib61] Pavli A., Maltezou H.C. (2021). COVID-19 vaccine passport for a safe resumption of travel. J. Travel Med..

[bib62] Pennycook G., McPhetres J., Zhang Y., Lu J.G., Rand D.G. (2020). Fighting COVID-19 misinformation on social media: experimental Evidence for a scalable accuracy-nudge intervention. Psychol. Sci..

[bib63] Plohl N., Musil B. (2020). Modeling compliance with COVID-19 prevention guidelines: the critical role of trust in science. Psychol., Health Med..

[bib64] Ravlin E.C., Liao Y., Morrell D.L., Au K., Thomas D.C. (2012). Collectivist orientation and the psychological contract: mediating effects of creditor exchange ideology. J. f Int. Business Stud..

[bib65] Rocklöv J., Sjödin H., Wilder-Smith A. (2020). COVID-19 outbreak on the Diamond Princess cruise ship: estimating the epidemic potential and effectiveness of public health countermeasures. J. Travel Med..

[bib66] Rowe F. (2020). Contact tracing apps and values dilemmas: a privacy paradox in a neo-liberal world. Int. J. Inf. Manag..

[bib67] Rowe F., Ngwenyama O., Richet J.-L. (2020). Contact-tracing apps and alienation in the age of COVID-19. Eur. J. Inf. Syst..

[bib68] Salathé M., Althaus C.L., Neher R., Stringhini S., Hodcroft E., Fellay J., Zwahlen M., Senti G., Battegay M., Wilder-Smith A., Eckerle I., Egger M., Low N. (2020). COVID-19 epidemic in Switzerland: on the importance of testing, contact tracing and isolation. Swiss Medical Weekly.

[bib69] Shahroz M., Ahmad F., Younis M.S., Ahmad N., Kamel Boulos M.N., Vinuesa R., Qadir J. (2021). COVID-19 digital contact tracing applications and techniques: a review post initial deployments. Transp. Eng..

[bib70] Sharun K., Tiwari R., Dhama K., Rabaan A.A., Alhumaid S. (2021). COVID-19 vaccination passport: prospects, scientific feasibility, and ethical concerns. Human Vaccines Immunother..

[bib71] Siegel S., John Castellan N. (2000).

[bib72] Son C., Hegde S., Smith A., Wang X., Sasangohar F. (2020). Effects of COVID-19 on college students’ mental health in the United States: interview survey study. J. Med. Internet Res..

[bib73] Sotis C., Allena M., Reyes R., Romano A. (2021). COVID-19 vaccine passport and international traveling: the combined effect of two nudges on Americans’ support for the pass. Int. J. Environ. Res. Public Health.

[bib74] Tencent (2020). Zhang Lijun, vice president of Tencent, said the number of people using health codes on wechat has exceeded 900 million this year. New.qq.com. https://new.qq.com/omn/20201228/20201228A0G1R100.html.

[bib75] Ukpabi D., Olaleye S., Karjaluoto H. (2020). Factors influencing tourists’ intention to use COVID-19 contact tracing app. Inf. Commun. Technol. Tourism.

[bib76] UNWHO (2020). Public health surveillance for COVID-19: interim guidance. http://Www.who.int.

[bib77] UNWHO (2020). WHO Director-General’s opening remarks at the media briefing on COVID-19-11 March 2020. http://Www.who.int.

[bib78] Urbaczewski A., Lee Y.J. (2020). Information Technology and the pandemic: a preliminary multinational analysis of the impact of mobile tracking technology on the COVID-19 contagion control. Eur. J. Inf. Syst..

[bib79] Villius Zetterholm M., Lin Y., Jokela P. (2021). Digital contact tracing applications during COVID-19: a scoping review about public acceptance. Informatics.

[bib80] Vokinger K.N., Nittas V., Witt C.M., Fabrikant S.I., von Wyl V. (2020). Digital health and the COVID-19 epidemic: an assessment framework for apps from an epidemiological and legal perspective. Swiss Med. Weekly.

[bib81] Wang T., Jia F. (2020). The impact of health QR code system on older people in China during the COVID-19 outbreak. Age Ageing.

[bib82] Weissberger A. (2020). China has more than 1.6 billion mobile subscribers (> China’s entire population!). Technol. Blog.

[bib83] Wiertz C., Banerjee A., Acar O.A., Ghosh A. (2020). Predicted adoption rates of contact tracing app configurations – insights from a choice-based conjoint study with a representative sample of the UK population. SSRN Electron. J..

[bib84] Wu C., Shi Z., Wilkes R., Wu J., Gong Z., He N., Xiao Z., Zhang X., Lai W., Zhou D., Zhao F., Yin X., Xiong P., Zhou H., Chu Q., Cao L., Tian R., Tan Y., Yang L., He Z. (2021). Chinese citizen satisfaction with government performance during COVID-19. J. Contempor. China.

[bib85] von Wyl V., Höglinger M., Sieber C., Kaufmann M., Moser A., Serra-Burriel M., Ballouz T., Menges D., Frei A., Puhan M.A. (2021). Drivers of acceptance of COVID-19 proximity tracing apps in Switzerland: panel survey analysis. JMIR Public Health Surveillance.

[bib86] Yasaka T.M., Lehrich B.M., Sahyouni R. (2020). Peer-to-Peer contact tracing: development of a privacy-preserving smartphone app. JMIR MHealth UHealth.

[bib87] Ying T., Wen J., Wang L. (2018). Language facilitation for outbound Chinese tourists: importance–performance and gap analyses of New Zealand hotels. J. Travel Tourism Market..

[bib88] Zhang Z., Schmude J. (2021). Understanding the travel constraints of potential Chinese tourists visiting Germany: experience from online travel community members. J. China Tourism Res..

[bib89] Zheng Y., Wang W., Zhong Y., Wu F., Zhu Z., Tham Y.-C., Lamoureux E., Xiao L., Zhu E., Liu H., Jin L., Liang L., Luo L., He M., Morgan I., Congdon N., Liu Y. (2021). A peer-to-peer live-streaming intervention for children during COVID-19 homeschooling to promote physical activity and reduce anxiety and Eye strain: cluster randomized controlled Trial. J. Med. Internet Res..

[bib90] Ziegel E.R. (1995). A step-by-step approach to using the SAS system for univariate and multivariate statistics. Technometrics.

